# Exploring the predictive capacity of smartphone-based digital phenotyping to monitor pain and physical quality of life in advanced cancer patients, family caregivers, and dyads

**DOI:** 10.3389/fpain.2026.1767157

**Published:** 2026-07-06

**Authors:** Kristen Allen-Watts, Andres Azuero, Kyungmi Lee, Erin R. Harrell, Erin Currie, Avery C. Bechthold, Sally Engler, Kayleigh Curry, Frank Puga, Natashia Bibriescas, Arif H. Kamal, Christine S. Ritchie, George Demiris, Alexi A. Wright, Marie A. Bakitas, Burel R. Goodin, J. Nicholas Odom

**Affiliations:** 1 Division of General Internal Medicine and Population Science, School of Medicine, University of Alabama at Birmingham, Birmingham, AL, United States; 2 School of Nursing, University of Alabama at Birmingham, Birmingham, AL, United States; 3 Frances Payne Bolton School of Nursing, Case Western Reserve University, Cleveland, OH, United States; 4 Division of Behavioral and Social Research, National Institute on Aging, National Institutes of Health, Bethesda, MD, United States; 5 College of Nursing, University of Tennessee at Knoxville, Knoxville, TN, United States; 6 Department of Medicine, Duke University School of Medicine, Durham, NC, United States; 7 Center for Optimal Aging and Serious Illness Research, Division of Palliative Care and Geriatric Medicine, Mass General Brigham and Harvard Medical School, Boston, MA, United States; 8 Department of Biobehavioral Health Sciences, School of Nursing & Department of Biostatistics, Epidemiology and Informatics, Perelman School of Medicine, University of Pennsylvania, Philadelphia, PA, United States; 9 Division of Population Sciences, Department of Medical Oncology, Dana-Farber Cancer Institute; Harvard Medical School, Boston, MA, United States; 10 Division of Gerontology, Geriatrics, and Palliative Care, School of Medicine, University of Alabama at Birmingham, Birmingham, AL, United States; 11 Center for Palliative and Supportive Care, University of Alabama at Birmingham, Birmingham, AL, United States; 12 Department of Anesthesiology, School of Medicine, Washington University in St Louis, St Louis, MO, United States

**Keywords:** advanced cancer, digital phenotyping, dyads, pain, family caregiver

## Abstract

**Introduction:**

Pain is among the most prevalent and distressing symptoms in advanced cancer, impairing physical, emotional, and social well-being. Management often requires support from family caregivers, whose own health and psychological well-being may also be adversely affected. This study examined the potential utility of digital phenotyping-moment-to-moment quantification of individual-level human behavior-to assess pain and physical quality of life (QOL) in patients with advanced cancer and their family caregivers.

**Methods:**

Patients with advanced cancer (*n* = 14) and their caregivers (*n* = 32) installed the Beiwe smartphone application, which enabled passive GPS data collection over 24 weeks. Raw GPS data were processed into daily mobility features and aggregated using biweekly moving averages and variability measures. Participants completed PROMIS measures of pain (intensity and interference) and physical QOL every 6 weeks. Within-person regression models were used to examine associations between changes in passive mobility features and changes in outcomes, with adjusted R² interpreted as effect size (small = 0.02, medium = 0.13, large = 0.26).

**Results:**

Caregiver GPS-derived mobility features predicted a large proportion of variance in patient pain intensity (R² = 0.31) and pain interference (R² = 0.32). Combined caregiver and patient mobility data predicted large variance in caregiver physical QOL (R² = 0.43) and medium-to-large variance in patient pain intensity (R² = 0.16) and pain interference (R² = 0.33). Patient mobility features alone predicted small variance in caregiver physical QOL (R² = 0.02). When examining patient data predicting patient outcomes, mobility features were associated with small variance in physical QOL (R² = 0.03), pain intensity (R² = 0.05), and pain interference (R² = 0.08).

**Discussion:**

These findings suggest that digital phenotyping may be a useful approach for predicting pain and physical QOL in advanced cancer, particularly when incorporating both patient and caregiver data. Further research is warranted to evaluate digital phenotyping as a novel method for monitoring symptoms and functional outcomes in advanced cancer care.

## 1 Introduction

Pain is one of the most prevalent and debilitating symptoms that people with advanced cancer experience (1), affecting more than 70% of patients in advanced stages of illness (2). Its physical toll is profound, pain limits mobility, disrupts daily activities such as eating, walking, and self-care, and contributes to fatigue and progressive functional decline (3). These impairments in physical function and overall physical well-being are central to the lived experience of advanced cancer (4).

Despite its prevalence and impact, pain remains frequently undertreated (5) The physical disability caused by pain often necessitates assistance from family caregivers, who can take on physically demanding tasks such as helping with mobility, functional support, and other daily activities (6). These responsibilities can lead to physical exhaustion and neglect of caregivers' own health (7). While emotional and psychological burdens are significant, the physical strain on caregiving in the context of pain is a critical and often underrecognized aspect of the caregiving experience (7, 8).

Pain complexity makes accurate assessment challenging. Traditional methods rely on self-report, which has several limitations and challenges. First, pain is dynamic and fluctuates over time, yet most self-report assessments are scheduled and episodic and may fail to accurately capture acute changes in pain, particularly in outpatient and home settings (9). Second, self-reported measures can be burdensome to administer and complete as one more task added to the daily routine (10). The limitations of pain and symptom self-report highlight the need for innovative, less intrusive alternative approaches to better reflect patient and caregivers lived experiences over the cancer treatment trajectory.

In response to these challenges, a limited number of dyadic home-based monitoring systems– most notably the Behavioral and Environmental Sensing and Intervention for Cancer (BESI-C) platform– have been developed to capture real-time symptom and behavioral data from patients with advanced cancer and their family caregivers within naturalistic home environments (11, 12). Although these systems demonstrate feasibility and provide contextual information about dyadic symptom experiences, they represent only one approach to capturing real-world cancer symptom dynamics. Digital phenotyping builds on this emerging body of work by using passively collected smartphone sensor data to provide a low-burden, scalable, and unobtrusive complementary approach to monitoring dyadic experiences over time.

Digital phenotyping is defined as the moment-by-moment quantification of the individual-level human phenotype *in situ* using data from personal digital devices such as smartphones and other wearables [e.g., global positioning system (GPS), accelerometer, gyroscope]. By passively and continuously collecting real-world behavioral data (13–15), digital phenotyping enables near real-time behavioral monitoring (15, 16) of domains as sleep patterns, mobility, speech, and social engagement. These passively derived indicators may offer meaningful insight into relevant patient-reported outcomes such as pain and physical quality of life in advanced cancer patients. For example, mobility patterns captured through GPS and accelerometry data may serve as indicators for functional capacity and physical well-being, both of which are closely tied to quality of life in this population. Similarly, reductions in mobility or changes in daily routines may signal pain exacerbations or increased symptom burden, as pain often constrains movement and participation in daily activities. At the dyadic level, caregiver mobility patterns may also reflect caregiving demands and patient needs, offering additional context for understanding patient and caregiving functioning.

Although digital phenotyping has shown promise in a number of health care contexts, its exploration in cancer has just begun to emerge (17–20). Further, there have been few reports on using digital phenotyping, particularly GPS data, which offers a unique lens into real-world mobility and location patterns in relation to pain and physical QOL, to incorporate passive data from both patients and caregivers as a dyadic pairing. To address these gaps, our study aimed to explore the potential utility of digital phenotyping as a method for assessing physical quality of life (QOL) and pain among patients with advanced cancer and their caregivers. By comparing patients and caregivers passively collected smartphone GPS data with traditional self-reported measures, we sought to evaluate the capacity for predicting pain using smartphone digital phenotyping.

## 2 Methods

### 2.1 Study design and setting

This was a prospective, longitudinal observational pilot feasibility study conducted between August 2021 and July 2023. Details of the study procedures have been described in detail elsewhere (21, 22). In brief, participants were recruited from outpatient oncology clinics at the University of Alabama at Birmingham (UAB) or via self-referral through Facebook advertisements using a professional digital marketing agency (High Level Marketing, Inc.). Upon initial contact, the project manager administered an additional screening assessment to verify eligibility prior to enrollment. After enrollment, participants installed the Beiwe digital phenotyping app (23) on their personal smartphones, which passively collected GPS data continuously over a 24-week period. Participants also completed surveys at baseline and every six weeks over the 24-week period. All eligible participants had to own a smartphone (Android or iOS). Eligible caregivers were 18 years or older and self-identified as an unpaid relative or friend providing regular support to a patient with cancer. Eligible patients were 18 years or older and diagnosed with advanced stage III or IV solid malignancies, or hematologic malignancies. Patient participants were undergoing active cancer treatment at the time of enrollment and were not enrolled in hospice. Because of this, some patients were unable or unwilling to participate. As a result, patient participation was encouraged but not required, and dyadic modeling was conducted when matched data were available. Participants received $25 for app installation and data transmission every six weeks, as well as $25 for each completed survey. The study was approved by the University of Alabama at Birmingham Institutional Review Board (IRB-300006575), and informed consent was obtained from both patient and caregiver.

### 2.2 The Beiwe Research Platform and smartphone App

Developed by the Onnela Lab at Harvard University, the Beiwe Research platform is an open-source system designed for high-resolution digital phenotyping using smartphones (23, 24). The platform includes a native app for both Android and iOS, which collects both active and passive data (23). Active data includes customizable surveys and audio recordings, while passive data includes GPS, accelerometer, gyroscope, call and text logs, and screen usage (23). The app is designed for minimal participant interaction, typically requiring only a system-generated user ID, password, and server IP address (23, 24). The backend infrastructure of Beiwe is hosted on Amazon Web Services (AWS). Researchers manage studies through a secure, web-based portal that allows full customization of sensor settings, sampling frequencies, survey content, and data upload parameters. Study configurations are stored in files, enabling easy replication and reproducibility (23, 24). Data is encrypted both in transit and at rest, and the system is designed to ensure that even the app cannot access its own stored data, maintaining strict privacy and security.

#### 2.2.1 Beiwe features used in this study

Although Beiwe supports a wide array of passive sensors, only a subset of these features was enabled for this study, and the configuration differed by smartphone operating system. On iOS devices, the Beiwe app was configured to collect accelerometer, GPS, and gyroscope, when available (i.e., when the device was on and not in idle mode). On Android devices, Beiwe was only able to collect accelerometer and GPS records for this study. While Beiwe can collect additional passive data (e.g., call/text logs), these features were intentionally disabled to reduce privacy concerns and because certain passive elements cannot be collected on iOS due to operating-system restrictions. Thus, the passive data used in this analysis consisted of GPS-derived location traces and device-based motion sensor signals.

The Beiwe app downloaded by participants was pre-programmed to collect GPS data, including coordinates for latitude, longitude, and altitude whenever the phone was in active motion or use rather than idle. To conserve battery life, sensor data was collected in 90 s bursts followed by 810 s off, resulting in approximately 6 min of GPS sampling per hour.

### 2.3 Measures

Caregiver and patient demographic data were self-reported.

#### 2.3.1 Participant-reported outcome (PRO) measures

Physical QOL was measured using the Patient-Reported Outcome Measurement Information System (PROMIS) Global Health-10*.* This 10-item short form and well-validated tool measures QOL using two subscales for mental and physical health (25–29). For the purposes of this analysis, we used only the physical health subscale. Responses are scored on a 5-point Likert-type scale, with higher scores reflecting better QOL (25–29). Standardized T-scores are generated, which have a mean of 50 and standard deviation of 10 in the U.S. general population (22). The PROMIS Global Health 10 has been widely used in oncology and palliative care research and demonstrates strong psychometric properties across diverse populations. The physical health subscale has shown high internal consistency and construct validity, making it a reliable and valid measure of physical well-being in individuals with serious illness (30).

Pain was measured using the PROMIS Pain Interference scale and the PROMIS Pain Intensity scale. The PROMIS Pain Interference short form evaluates the extent to which pain hinders engagement in daily activities and affects social, cognitive, emotional, and physical functioning. Items are rated on a 5-point Likert-type scale, with higher scores indicating greater interference of pain on daily life. The PROMIS Pain Intensity scale consists of 3 items assessing the respondent's average level of pain, intensity of pain, and current pain over the past seven days, also rated on a 5-point scale. Both measures are converted to standardized T-scores relative to the U.S. general population.

PRO measures were administered at baseline and every 6 weeks for 24 weeks, a schedule that provides sufficient time to capture clinically meaningful symptom changes while minimizing participant burden.

### 2.4 Analysis

To address missing or incomplete GPS data, we required a minimum of 20% of expected daily GPS coverage for a day to be considered valid. We then summarized valid daily data into biweekly moving averages to reduce noise. Among participants who contributed GPS data, the median number of days with usable data was 141 out of 168, with an average of 98.2 min of GPS data per day (≈81.8% of the daily target).

Raw GPS data were pre-processed using R scripts provided by the Beiwe platform (21, 31) to generate daily mobility-derived features (e.g., distance traveled, time at home, circadian routine). A full description of the derived GPS mobility features has been detailed in a previous paper (22). To capture patterns of mobility over time, biweekly averages and standard deviations were computed, yielding 28 summary features.

To reduce the high dimensionality and redundancy among these correlated mobility features, we conducted dimension reduction via principal components analysis (PCA). Principal components are new variables, computed as linear combinations of the original correlated features, that capture the maximum variance using fewer dimensions. Six components were retained based on eigenvalues >1. This step was essential for simplifying the high-dimensional GPS-derived mobility features, such as location variability, time spent at home, and travel patterns, into a smaller set of uncorrelated components that preserved the most meaningful variation in the data.

Importantly, each principal component score represents a composite pattern of mobility behaviors derived from the underlying passive sensor data. Thus, associations between principal component scores and PRO should be interpreted as relationships between broader mobility behavior patterns (captured via passive sensing) and outcomes, such as pain and physical quality of life. Details of preprocessing, feature derivation, and PCA validation are reported elsewhere (21).

#### 2.4.1 Linking GPS-derived components with PRO measures

Each PRO assessment was linked to the biweekly GPS summary window that immediately preceded the PRO date, ensuring that mobility features reflected the behavioral period leading up to each symptom report.

To examine how changes in mobility related to symptom changes, PRO assessments were matched by date with the corresponding biweekly principal component scores from the patient and their caregiver. For within-person analyses, PRO change scores served as outcomes in separate caregiver and patient regression models. Both individual-level and dyad-level principal component scores were entered as predictors. Details of correlation estimates between the change in PROs and change in principal component scores were previously reported in a Supplemental Table from a prior published analysis (22).

PRO change scores were calculated as the difference between consecutive 6-week assessments (e.g., week 12 minus week 6). GPS-derived principal component scores were similarly linked to each assessment period using the biweekly mobility summary window immediately preceding each PRO assessment. Sensor data change scores were then calculated as the difference between these temporally matched biweekly summaries across consecutive assessment periods.

For the regression models, the overall measure of effect size was adjusted-R^2^, representing the proportion of variance in the outcome explained by our predictors. A 95% confidence interval for adjusted-R^2^ was computed using cluster Jackknife resampling. For individual predictors (i.e., principal component scores), effect sizes were estimated using Omega^2^, interpreted as small = 0.01, medium = 0.06, and large = 0.14.

## 3 Results

### 3.1 Participant demographic and clinical data

Among the 58 participants who consented and successfully downloaded the app (40 caregivers, 18 patients), usable data were obtained from 46 individuals [32 caregivers, 14 patients (resulting in dyadic data for 14 pairs)]. Participants were included in the analytic sample if they had at least one valid PRO assessment that could be matched to a corresponding GPS mobility window. Demographic characteristics of caregivers (*N* = 32), patients (*N* = 14), and matched pairs are shown in Table 1. Most caregivers were White (78.1%), female (84.4%), worked full or part time (72.5%), and had a college or graduate degree (40.6%). Most patients were White (92.9%), female (57.1%), retired (28.6%) or on disability (28.6%), and had a college or graduate degree (50%). The mean age of caregivers was 49.4 years (SD 12.8). The mean age of patients was 59.1 years (SD 8.7). Caregivers were most often a spouse (43.8%) or adult child (31.2%) of the patient. On average, PROMIS Global Physical T scores among family caregivers were 0.40–0.50 standard deviation below the general adult population mean. Patients had lower scores, roughly 0.60–0.70 standard deviation below the general population mean. Among the matched caregiver sample, the observed standard deviation indicates slightly greater variability in physical health. Among patients, PROMIS Pain Intensity T scores were, on average, 18% of a standard deviation above the general adult population mean, and the average PROMIS Pain Interference T score was 39% of a standard deviation above the general adult population mean. Regression analyses are shown in Table 2.

**Table 1:** Caregiver and patient demographics.

Characteristic	Family Caregivers (*N* = 32)	Patients (*N* = 14)	Matched Family Caregivers (*N* = 14)
	Number (%) or Mean (standard deviation)		
Age	49.4 (12.8)	59.1 (8.7)	52.0 (14.5)
Gender			
Female	27 (84.4)	8 (57.1)	10 (71.4)
Male	5 (15.6)	6 (42.9)	4 (28.6)
Race			
White	25 (78.1)	13 (92.9)	13 (92.9)
Black/African American	7 (21.9)	1 (7.1)	1 (7.1)
Hispanic/Latino/a/x			
No	30 (93.5)	14 (100.0)	14 (100.0)
Yes	2 (6.5)	0 (0.0)	0 (0.0)
Employment			
Full/part-time	20 (72.5)	3 (21.4)	11 (78.6)
Retired	2 (6.2)	4 (28.6)	1 (7.1)
Homemaker	5 (15.6)	3 (21.4)	1 (7.1)
Disability/Other	5 (15.6)	4 (28.6)	1 (7.1)
Education			
8th grade or less	1 (3.1)	0 (0.0)	1 (7.1)
High school graduate or GED	8 (25.0)	3 (21.4)	2 (14.3)
Some college or technical school	10 (31.2)	4 (28.6)	4 (28.6)
College graduate/Graduate degree	13 (40.6.)	7 (50.0)	7 (50.0)
Caregiver-patient relationship (The patient is the caregiver's…)			
Spouse	14 (43.8)	n/a	10 (71.4)
Parent	10 (31.2)	n/a	2 (14.3)
Other	7 (21.9)	n/a	1 (7.1)
Missing	1 (3.1)	n/a	1 (7.1)
Cancer type^a^			
Gastrointestinal (e.g., Colon/Rectal, Head/Neck)	10 (31.2)	6 (42.8)	6 (42.8)
Genitourinary (e.g., Prostate, Bladder/Kidney)	7 (21.9)	4 (28.6)	4 (28.6)
Thoracic (e.g., Lung)	4 (12.5)	1 (7.1)	1 (7.1)
Breast	3 (9.4)	1 (7.1)	1 (7.1)
Other	8 (25.0)	2 (14.3)	2 (14.3)
PROMIS Global Health-10			
Physical QoL T	45.6 (10.9)	43.7 (8.9)	49.0 (9.3)
PROMIS Pain Intensity T	N/A	51.8 (10.6)	N/A
PROMIS Pain Interference T	N/A	53.9 (9.1)	N/A

N/A: not applicable.

PROMIS Global Health-10: T- scores ≤ 50 suggests below-average physical health.

PROMIS Pain Intensity: Higher T-scores indicate greater pain intensity; T-score of 50 represents the average pain level in general population.

PROMIS Pain Interference: Higher T-scores indicate more interference from pain; T-score of 50 is the mean for the general population.

a Cancer type and stage for caregiver refers to care recipient.

**Table 2:** Measures of effect (Omega^2^) in linear regressions of within-person changes in outcomes predicted by changes smartphone biweekly feature principal components.

Outcomes	Omega^2^ for 6 Principal Components - Caregivers						Omega^2^ for 6 Principal Components - Patients						Adjusted R^2^	95% CI		Multiple R
	1	2	3	4	5	6	1	2	3	4	5	6		Lower	Upper	
Caregiver smartphone data predicting CAREGIVER OUTCOMES (57 instances of change from *n* = 27 caregivers)																
PROMIS- Physical Function	0	0	0	0	0.02	0	-	-	-	-	-	-	0	0	0.02	0
Patient smartphone data predicting CAREGIVER OUTCOMES (29 instances of change from *n* = 12 dyads)																
PROMIS- Physical Function		-	-	-	-	-	0	0.06	0	0.04	0	0	0.02	0	0.63	0.14
Caregiver and Patient smartphone data predicting CAREGIVER OUTCOMES (23 instances of change from *n* = 11 dyads)																
PROMIS- Physical Function	0	0.06	0	0.11	0.03	0	0	0.04	0.01	0.18	0.01	0.03	.43	0	0.93	0.66
Caregiver smartphone data predicting PATIENT OUTCOMES (32 instances of change from *n* = 14 dyads)																
PROMIS- Physical Function	0	0	0	0.04	0	0	-	-	-	-	-	-	0	0	0.2	0
PROMIS-Pain Intensity	0	0.1	0.03	0.24	0	0	-	-	-	-	-	-	0.31	0	0.74	0.56
PROMIS-Pain Interference	0	0.19	0.01	0.18	0	0	-	-	-	-	-	-	0.32	0	0.82	0.57
Patient smartphone data predicting PATIENT OUTCOMES (31 instances of change from *n* = 13 patients)																
PROMIS- Physical Function	-	-	-	-	-	-	0	0.01	0	0.03	0	0.05	0.03	0	0.47	0.17
PROMIS-Pain Intensity	-	-	-	-	-	-	0	0.09	0	0	0.01	0.04	0.05	0	0.7	0.22
PROMIS-Pain Interference	-	-	-	-	-	-	0.01	0.05	0	0	0	0.06	0.08	0	0.77	0.28
Caregiver and Patient smartphone data predicting PATIENT OUTCOMES (26 instances of change from *n* = 11 dyads)																
PROMIS- Physical Function	0.03	0	0	0.02	0	0	0	0	0	0	0	0	0	0	0.61	0
PROMIS-Pain Intensity	0	0.23	0	0.19	0	0	0	0	0	0	0	0	0.16	0	0.98	0.40
PROMIS-Pain Interference	0	0.18	0	0.25	0	0	0	0	0	0.06	0	0	0.33	0	0.97	0.57

Adjusted R^2^ as effect size: small = 0.02; medium = 0.13; large = 0.26.

Omega^2^ as effect size: small = 0.01; medium = 0.06; large = 0.14.

#### 3.1.1 Smartphone predictors of caregiver outcomes

When caregiver smartphone data were used to predict caregiver physical function (*n* = 27), the adjusted-R^2^s was zero, indicating no meaningful association. However, when patient smartphone data were used to predict caregiver physical function (*n* = 12), the adjusted- R^2^ was small to negligible magnitude (0.02). When caregiver and patient smartphone data were combined (*n* = 11), the adjusted-R^2^s for physical function was large (0.43). At the predictor level (Table 2), Omega^2^ values for principal component scores ranged from small to moderate across models.

#### 3.1.2 Smartphone predictors of patient outcomes

For caregiver smartphone data predicting patient outcomes (*n* = 14), the adjusted-R^2^s was zero for physical function (0); but large for pain intensity (0.31) and pain interference (0.32). when patient smartphone data were used (*n* = 13), the adjusted-R^2^ values were small for physical function (0.03) and small to medium for pain intensity (0.05) and pain interference (0.08). When caregiver and patient smartphone data were combined (*n* = 11), adjusted-R^2^ remained zero for physical function (0), medium to large for pain intensity (0.16), and large for pain interference (0.33). Across these models, individual principal components demonstrated small to moderate effect sizes based on Omega^2^ (Table 2).

## 4 Discussion

This study explored the potential utility of digital phenotyping as a method for assessing pain and physical QOL in patients with advanced cancer and their family caregivers. Several key findings emerged from this exploratory analysis. First, our results suggest that caregiver activity patterns and daily movement behaviors, captured through passive smartphone GPS data, may be associated with patient pain self-reports. Specifically, caregiver smartphone data— in this case the variance over time in their smartphone GPS patterns– accounted for a large proportion of variance in patient pain intensity (adjusted R^2^ = 0.31) and pain interference (adjusted R^2^ = 0.32). Consistent with these findings, predictor-level analyses indicated that several principal components demonstrated small to moderate effect sizes based on Omega^2^, suggesting that specific mobility-derived behavioral features contributed to these associations. These results contribute to the growing literature on the role of caregivers in the patient cancer pain experience (32, 33), extending prior work by suggesting that caregiver mobility and behavioral patterns may serve as indirect indicators of patient burden.

One potential explanation for these findings is that passive mobility patterns may serve as proximal behavioral indicators of evolving symptoms burden and functional status. In advanced cancer, worsening pain and reduced physical quality of life may manifest through changes in movement, time spent at home, disrupted daily routines, and increased caregiver involvement. Consequently, short-term GPS-derived mobility patterns may reflect broader behavioral adaptations occurring alongside clinically meaningful symptom changes over time.

Whereas prior literature has focused broadly on caregivers' roles in cancer pain management (34–37), our findings suggest that pain dimensions, especially pain intensity and pain interference, may help explain when and how caregivers become more engaged. These dimensions were the patient outcomes most strongly predicted by caregiver mobility patterns in our analyses, indicating that caregiver behavioral signals may align more closely with certain aspects of patient pain than others.

A key observation that raises an important direction going forward is the potential added value of integrating both patient and caregiver data to enhance predictive power (i.e., changes in physical health and symptoms like pain). When combining both caregiver and patient data, the predictive power appeared to be enhanced. Specifically, the combined caregiver and patient data explained a large proportion of variance in caregiver physical QOL scores (adjusted R^2^ = 0.43), and medium to large variance in patient pain intensity (adjusted R^2^ = 0.16) and interference (adjusted R^2^ = 0.33). These findings suggest that dyadic data may offer a more comprehensive and sensitive approach to monitoring caregiver general health and well-being as well as symptom burden among advanced cancer patients. The predictive value of combined data highlights the interdependent nature of patient-caregiver relationships in advanced cancer care, where changes in one individual's behavior or health status may reflect or influence outcomes in the other (38, 39). This further supports the growing recognition of dyadic frameworks in symptom management and highlights the potential of digital phenotyping to capture these relationships in real-world settings. Importantly, this approach to passive monitoring may also afford models of care in which clinicians can remotely monitor early signs of worsening pain and other symptoms through data already generated by patients' and family caregivers' smartphones.

In contrast, patient data alone showed limited predictive utility for caregiver outcomes, with only a small proportion of variance explained in caregiver physical QOL (adjusted R^2^ = 0.02). Similarly, when patient data were used to predict their own outcomes, the effect sizes were small across all measures: physical QOL (adjusted R^2^ = 0.03), pain intensity (adjusted R^2^ = 0.05), and pain interference (adjusted R^2^ = 0.08). These findings suggest that passive data from patients alone may provide an incomplete picture of fluctuations in overall health and pain experiences, highlighting the potential value of incorporating caregiver data to better understand acute changes in PROs, such as pain and physical QOL, in real-world settings. This may, in part, reflect the very small sample sizes in these analyses, which could influence the extent to which passive data captures meaningful variation.

### 4.1 Why patient mobility data may not strongly predict caregiver outcomes

Although patient mobility declines as illness progresses, this decline may not necessarily produce corresponding changes in caregiver mobility patterns. In our study, the majority of family caregivers lived in a shared residence with the patient. Prior cancer caregiving research shows that family caregivers, especially co-residing spouses and family members, provide most cancer-related support within the home (40, 41). Consequently, even when patient care needs increase, caregivers' mobility patterns may remain stable because the most intensive caregiving tasks occur in the household. However, because this study is exploratory and included small sample sizes, these conjectures will need to be tested in future studies.

Many of the contributors to caregiver burden in cancer, such as sustained vigilance for treatment complications, emotional strain, complex decision-making, and disruptions to sleep (42, 43), also occur in the home and do not require reductions in geographic movement. Cancer caregiving typically involves physically and psychosocially demanding in-home responsibilities, such as managing pain, nausea, fatigue, and medication timing, which are not directly reflected in spatial movement patterns. Thus, the limited predictive utility of patient mobility for caregiver physical quality of life may reflect a mismatch between the behavioral signal captured (geolocation variability) and the multidimensional construct of caregiver well-being.

This study has several limitations that warrant consideration. First, it is important to acknowledge that our sample size was small, limiting statistical power and reducing our ability to detect subtle associations between patient mobility and caregiver outcomes. Effect sizes should thus be interpreted as exploratory signals rather than definitive estimates of predictive utility. Future studies are needed that include larger sample sizes to more definitively validate this approach to pain and physical health monitoring. Second, the sample lacked diversity in race/ethnicity and gender, potentially restricting the applicability of findings to broader populations. This limitation is particularly important given the disproportionate burden of advanced cancer among racial and ethnic minorities (44–47), as well as well-documented disparities in pain experiences and treatment (48, 49). Research has shown that Black patients often report higher pain intensity and greater disability, yet receive less appropriate pain management, partly due to implicit bias and false beliefs about pain tolerance (48). Although intentional efforts were made to recruit a diverse sample, future studies should prioritize larger and more demographically representative cohorts to enhance external validity across different demographic groups. Since we did not assess baseline digital literacy levels of participants, we may have excluded patients or caregivers who do not have access to or are not comfortable using a smartphone. Third, participation required access to a smartphone and a certain level of comfort and proficiency with mobile apps. This may have excluded individuals who did not have a smartphone, were not technologically proficient, or were experiencing cognitive impairments due to cancer or its treatment, thereby limiting the generalizability of the findings. As a feasibility study, this work was intended to explore the potential of digital phenotyping in a real-world context; however, future studies should consider strategies to reduce technology-related barriers, expand accessibility to more diverse populations, and leverage mixed effects models to examine within-person changes in patient-reported outcomes over time. Fourth, although this study used validated PROs, these tools rely on retrospective self-report. This approach may be particularly challenging for patients with advanced cancer due to symptom burden and potential cognitive impairment, introducing recall bias and limiting the ability to capture dynamic fluctuations in pain and functioning. Fifth, this study utilized only GPS-derived mobility features from the available passive smartphone data. Our prior work suggests that participants generally report low privacy concerns related to GPS-based passive monitoring, supporting its acceptability as a low-burden sensing modality. Additionally, many meaningful behavioral features can be derived from GPS data alone. However, the inclusion of additional passive sensing modalities, such as accelerometry, screen usage, sleep/wake patterns, and device interaction data, may have provided a more comprehensive representation of behavioral and functional status. Incorporating these multimodal data streams in future studies may improve the ability to detect changes in pain and physical quality of life by capturing a broader range of behavioral signals.

Finally, we did not collect data on the specific roles that caregiver participants played in assisting patients with their pain management or their physical well-being (e.g., medication administration, symptom monitoring, exercise/mobility support, transportation). Future studies might consider including a caregiver role inventory to better isolate how discrete caregiving activities relate to digital phenotypes, and analyses should be stratified based on cancer severity and cancer type to account for potential differences in caregiving needs and patterns.

## 5 Conclusion

In summary, this exploratory study demonstrates the potential of digital phenotyping to assess pain and physical QOL in patients with advanced cancer and their caregivers using passively collected smartphone data. Key findings highlight the unique value of caregiver data in predicting patient pain outcomes, and the enhanced predictive utility achieved when combining patient and caregiver data. These results underscore the importance of dyadic approaches in symptom monitoring and intervention design, particularly in the context of advanced cancer where health experiences are deeply interdependent. Our study highlights the importance of capturing fluctuations in symptom burden, reinforcing the need to incorporate caregiver perspectives and behaviors into digital health models. Future research should build on these exploratory findings by using larger, more diverse samples and integrating real-time digital data collection methods. Doing so may help refine digital phenotyping models and improve their ability to detect clinically meaningful patterns that reflect both individual and relational health dynamics.

## Data Availability

The raw data supporting the conclusions of this article will be made available by the authors upon reasonable request, without undue reservation.

## References

[1] Cooley ME. Point-of-care clinical decision support for cancer symptom management: results of a group randomized trial. ASCO. (2014) 32. 10.1200/jco.2014.32.31_suppl.1

[2] Dalal S Bruera E. Pain management for patients with advanced cancer in the opioid epidemic era. Am Soc Clin Oncol Educ Book. (2019) 39:24–35. 10.1200/EDBK_100020 31099619

[3] Hadjiat Y Arendt-Nielsen L. Digital health in pain assessment, diagnosis, and management: overview and perspectives. Front Pain Res. (2023) 4:1097379. 10.3389/fpain.2023.1097379 PMC10149799 37139342

[4] Snijders R Brom L Theunissen M van den Beuken-van Everdingen M. Update on prevalence of pain in patients with cancer 2022: a systematic literature review and meta-analysis. Cancers (Basel). (2023) 15:591. 10.3390/cancers15030591 36765547 PMC9913127

[5] DuBenske LL. Advanced cancer: palliative, end of life, and bereavement care. In: Hesse BW, Ahern DK, Beckjord E, editors. *Oncology Informatics: Using Health Information Technology to Improve Processes and Outcomes in Cancer*. London: Academic Press (2016). p. 181–203.

[6] Litzelman K. Caregiver well-being and the quality of cancer care. Semin Oncol Nurs. (2019) 35:348–53. 10.1016/j.soncn.2019.06.006 31229346 PMC6728914

[7] Fu M Shen J Gu C Oliveira E Shinchuk E Isaac H. The pain intervention & digital research program: an operational report on combining digital research with outpatient chronic disease management. Front Pain Res. (2024) 5:1327859. 10.3389/fpain.2024.1327859 PMC10869590 38371228

[8] Ye Y Cardoso D de M Kayahara GM Bernabé DG. A pilot study to improve pain phenotyping in head and neck cancer patients. Front Pain Res. (2023) 4:1146667. 10.3389/fpain.2023.1146667 PMC10211332 37251594

[9] Murali KP Kang JA Bronstein D McDonald MV King L Chastain AM. Measuring palliative care-related knowledge, attitudes, and confidence in home health care clinicians, patients, and caregivers: a systematic review. J Palliat Med. (2022) 25:1579–98. 10.1089/jpm.2021.0580 35704053 PMC9639230

[10] Campbell R Ju A King MT Rutherford C. Perceived benefits and limitations of using patient-reported outcome measures in clinical practice with individual patients: a systematic review of qualitative studies. Qual Life Res. (2022) 31:1597–620. 10.1007/s11136-021-03003-z 34580822

[11] Ostovari M Crimp N Ratcliffe SJ LeBaron V. Defining dyadic cancer pain concordance using participant-initiated interactions with a remote health monitoring system. JAMIA Open. (2025) 8:ooaf088. 10.1093/jamiaopen/ooaf088 40862001 PMC12373113

[12] LeBaron V Alam R Bennett R Blackhall L Gordon K Hayes J. Deploying the behavioral and environmental sensing and intervention for cancer smart health system to support patients and family caregivers in managing pain: feasibility and acceptability study. JMIR Cancer. (2022) 8:e36879. 10.2196/36879 35943791 PMC9399893

[13] Montag C Baumeister H. Digital Phenotyping and Mobile Sensing. Cham: Springer International Publishing (2023). 10.1007/978-3-030-98546-2

[14] Ensari I Caceres BA Jackman KB Suero-Tejeda N Shechter A Odlum ML. Digital phenotyping of sleep patterns among heterogenous samples of Latinx adults using unsupervised learning. Sleep Med. (2021) 85:211–20. 10.1016/j.sleep.2021.07.023 34364092 PMC8439405

[15] Sameh A Rostami M Oussalah M Korpelainen R Farrahi V. Digital phenotypes and digital biomarkers for health and diseases: a systematic review of machine learning approaches utilizing passive non-invasive signals collected via wearable devices and smartphones. Artif Intell Rev. (2024) 58:66. 10.1007/s10462-024-11009-5

[16] Lee JS Browning E Hokayem J Albrechta H Goodman GR Venkatasubramanian K. Smartphone and wearable device-based digital phenotyping to understand substance use and its syndemics. J Med Toxicol. (2024) 20:205–14. 10.1007/s13181-024-01000-5 38436819 PMC10959908

[17] Jenciūtė G Kasputytė G Bunevičienė I Korobeinikova E Vaitiekus D Inčiūra A. Digital phenotyping for monitoring and disease trajectory prediction of patients with cancer: protocol for a prospective observational cohort study. JMIR Res Protoc. (2023) 12:e49096. 10.2196/49096 37815850 PMC10599285

[18] Wright AA Raman N Staples P Schonholz S Cronin A Carlson K. The HOPE pilot study: harnessing patient-reported outcomes and biometric data to enhance cancer care. JCO Clin Cancer Inform. (2018) 2:1–12. 10.1200/CCI.17.00149 30652585 PMC6556148

[19] Panda N Solsky I Huang EJ Lipsitz S Pradarelli JC Delisle M. Using smartphones to capture novel recovery metrics after cancer surgery. JAMA Surg. (2020) 155:123. 10.1001/jamasurg.2019.4702 31657854 PMC6820047

[20] Low CA. Harnessing consumer smartphone and wearable sensors for clinical cancer research. NPJ Digit Med. (2020) 3:140. 10.1038/s41746-020-00351-x 33134557 PMC7591557

[21] Odom JN Lee K Currie ER Allen-Watts K Harrell ER Bechthold AC. Feasibility and acceptability of collecting passive smartphone data for potential use in digital phenotyping among family caregivers and patients with advanced cancer. JCO Clin Cancer Inform. (2025) 9:e2400201. 10.1200/CCI-24-00201 39746166 PMC11706344

[22] Odom JN Lee K Harrell ER Watts KA Bechthold AC Engler S. Associations between smartphone GPS data and changes in psychological health and burden outcomes among family caregivers and patients with advanced cancer: an exploratory longitudinal cohort study. BMC Cancer. (2025) 25:614. 10.1186/s12885-025-14009-y 40186196 PMC11971861

[23] Torous J Kiang MV Lorme J Onnela J-P. New tools for new research in psychiatry: a scalable and customizable platform to empower data driven smartphone research. JMIR Ment Health. (2016) 3:e16. 10.2196/mental.5165 27150677 PMC4873624

[24] Harvard T.H. Chan School of Public Health. Digital Phenotyping and Beiwe Research Platform. Onnela Lab Department of Biostatistics. Available online at: https://hsph.harvard.edu/research/onnela-lab/digital-phenotyping-and-beiwe-research-platform/ (Accessed August 14, 2025)

[25] Hahn EA Beaumont JL Pilkonis PA Garcia SF Magasi S DeWalt DA. The PROMIS satisfaction with social participation measures demonstrated responsiveness in diverse clinical populations. J Clin Epidemiol. (2016) 73:135–41. 10.1016/j.jclinepi.2015.08.034 26931288 PMC4902758

[26] Cella D Lai J-S Jensen SE Christodoulou C Junghaenel DU Reeve BB. PROMIS Fatigue item bank had clinical validity across diverse chronic conditions. J Clin Epidemiol. (2016) 73:128–34. 10.1016/j.jclinepi.2015.08.037 26939927 PMC4902759

[27] Askew RL Cook KF Revicki DA Cella D Amtmann D. Evidence from diverse clinical populations supported clinical validity of PROMIS pain interference and pain behavior. J Clin Epidemiol. (2016) 73:103–11. 10.1016/j.jclinepi.2015.08.035 26931296 PMC4957699

[28] Cook KF Jensen SE Schalet BD Beaumont JL Amtmann D Czajkowski S. PROMIS Measures of pain, fatigue, negative affect, physical function, and social function demonstrated clinical validity across a range of chronic conditions. J Clin Epidemiol. (2016) 73:89–102. 10.1016/j.jclinepi.2015.08.038 26952842 PMC5131708

[29] Schalet BD Hays RD Jensen SE Beaumont JL Fries JF Cella D. Validity of PROMIS physical function measured in diverse clinical samples. J Clin Epidemiol. (2016) 73:112–8. 10.1016/j.jclinepi.2015.08.039 26970039 PMC4968197

[30] Hays RD Schalet BD Spritzer KL Cella D. Two-item PROMIS® global physical and mental health scales. J Patient Rep Outcomes. (2017) 1(1):2. 10.1186/s41687-017-0003-8 PMC5934936 29757325

[31] Onnela Lab. Beiwe-Analysis. Available online at: https://github.com/onnela-lab/Beiwe-Analysis (Accessed October 1, 2025)

[32] Filipponi C Chichua M Masiero M Mazzoni D Pravettoni G. Cancer pain experience through the Lens of patients and caregivers: mixed methods social Media study. JMIR Cancer. (2023) 9:e41594. 10.2196/41594 37399067 PMC10365594

[33] Porter LS Samsa G Steel JL Hanson LC LeBlanc TW Bull J. Caregiver-guided pain coping skills training for patients with advanced cancer: background, design, and challenges for the CaringPals study. Clinical Trials. (2019) 16:263–72. 10.1177/1740774519829695 30782014 PMC6533140

[34] Chi N-C Nakad L Han S Washington K Hagiwara Y Riffin C. Family Caregivers' Challenges in cancer pain management for patients receiving palliative care. Am J Hosp Palliat Care. (2023) 40:43–51. 10.1177/10499091221094564 35503240 PMC10201988

[35] Chi N-C Barani E Fu Y-K Nakad L Gilbertson-White S Herr K. Interventions to support family caregivers in pain management: a systematic review. J Pain Symptom Manage. (2020) 60:630–656.e31. 10.1016/j.jpainsymman.2020.04.014 32339651 PMC7483228

[36] Han CJ Chi N-C Han S Demiris G Parker-Oliver D Washington K. Communicating caregivers' challenges with cancer pain management: an analysis of home hospice visits. J Pain Symptom Manage. (2018) 55:1296–303. 10.1016/j.jpainsymman.2018.01.004 29360571 PMC5899943

[37] Dave R Friedman S Miller-Sonet E Moore T Peterson E Fawzy Doran J. Identifying and addressing the needs of caregivers of patients with cancer: evidence on interventions and the role of patient advocacy groups. Future Oncology. (2024) 20:2589–602. 10.1080/14796694.2024.2387526 39329173 PMC11534103

[38] Kershaw T Ellis KR Yoon H Schafenacker A Katapodi M Northouse L. The interdependence of advanced cancer patients' and their family caregivers' mental health, physical health, and self-efficacy over time. Ann Behav Med. (2015) 49:901–11. 10.1007/s12160-015-9743-y 26489843 PMC4825326

[39] Ketcher D Otto AK Vadaparampil ST Heyman RE Ellington L Reblin M. The psychosocial impact of spouse-caregiver chronic health conditions and personal history of cancer on well-being in patients with advanced cancer and their caregivers. J Pain Symptom Manage. (2021) 62:303–11. 10.1016/j.jpainsymman.2020.12.008 33348028 PMC8213866

[40] Longacre ML Hunt GG Kent EE Weber-Raley L. Cancer caregiving in the U.S. An intense, episodic, and challenging care experience. (2016).

[41] National Cancer Institute. Cancer caregiving. Available online at: https://healthcaredelivery.cancer.gov/caregiving/ (Accessed June 25, 2026).

[42] Akter J Konlan KD Nesa M Ispriantari A. Factors influencing cancer patients' Caregivers' Burden and quality of life: an integrative review. Heliyon. (2023) 9:e21243. 10.1016/j.heliyon.2023.e21243 38027739 PMC10643105

[43] Rezaei M Keyvanloo Shahrestanaki S Mohammadzadeh R Aghili MS Rajabi M Abbasi M. Caregiving consequences in cancer family caregivers: a narrative review of qualitative studies. Front Public Health. (2024) 12:1334842. 10.3389/fpubh.2024.1334842 38584929 PMC10997218

[44] CDC. The Centers for Disease Control and Prevention. Cancer and African American People. Cancer (2025). Available online at: https://www.cdc.gov/cancer/health-equity/african-american.html (Accessed August 20, 2025)

[45] Özdemir BC Dotto G-P. Racial differences in cancer susceptibility and survival: more than the color of the skin? Trends Cancer. (2017) 3:181–97. 10.1016/j.trecan.2017.02.002 28718431 PMC5518637

[46] Njoku A Sawadogo W Frimpong P. Racial and ethnic disparities in cancer occurrence and outcomes in rural United States: a scoping review. Cancer Control. (2024) 31:10732748241261558. 10.1177/10732748241261558 38857181 PMC11165954

[47] Zavala VA Bracci PM Carethers JM Carvajal-Carmona L Coggins NB Cruz-Correa MR. Cancer health disparities in racial/ethnic minorities in the United States. Br J Cancer. (2021) 124:315–32. 10.1038/s41416-020-01038-6 32901135 PMC7852513

[48] Schoenthaler A Williams N. Looking beneath the surface: racial bias in the treatment and management of pain. JAMA Netw Open. (2022) 5:e2216281. 10.1001/jamanetworkopen.2022.16281 35679049

[49] Allen-Watts K Sims AM Buchanan TL DeJesus DJB Quinn TL Buford TW. Sociodemographic differences in pain medication usage and healthcare provider utilization among adults with chronic low back pain. Front Pain Res. (2022) 2:806310. 10.3389/fpain.2021.806310 PMC8915740 35295517

